# Essential Role of TGF-β/Smad Pathway on Statin Dependent Vascular Smooth Muscle Cell Regulation

**DOI:** 10.1371/journal.pone.0003959

**Published:** 2008-12-17

**Authors:** Juan Rodríguez-Vita, Eva Sánchez-Galán, Beatriz Santamaría, Elsa Sánchez-López, Raquel Rodrigues-Díez, Luís Miguel Blanco-Colio, Jesús Egido, Alberto Ortiz, Marta Ruiz-Ortega

**Affiliations:** 1 Cellular Biology in Renal Diseases Laboratory, Fundación Jiménez Díaz, Universidad Autónoma Madrid, Madrid, Spain; 2 Vascular Research Laboratory, Fundación Jiménez Díaz, Universidad Autónoma Madrid, Madrid, Spain; 3 Dialysis Unit, Fundación Jiménez Díaz, Universidad Autónoma Madrid, Madrid, Spain; University of Giessen Lung Center, Germany

## Abstract

**Background:**

The 3-hydroxy-3-methylglutaryl CoA reductase inhibitors (also called statins) exert proven beneficial effects on cardiovascular diseases. Recent data suggest a protective role for Transforming Growth Factor-β (TGF-β) in atherosclerosis by regulating the balance between inflammation and extracellular matrix accumulation. However, there are no studies about the effect of statins on TGF-β/Smad pathway in atherosclerosis and vascular cells.

**Methodology:**

In cultured vascular smooth muscle cells (VSMCs) statins enhanced Smad pathway activation caused by TGF-β. In addition, statins upregulated TGF-β receptor type II (TRII), and increased TGF-β synthesis and TGF-β/Smad-dependent actions. In this sense, statins, through Smad activation, render VSMCs more susceptible to TGF-β induced apoptosis and increased TGF-β-mediated ECM production. It is well documented that high doses of statins induce apoptosis in cultured VSMC in the presence of serum; however the precise mechanism of this effect remains to be elucidated. We have found that statins-induced apoptosis was mediated by TGF-β/Smad pathway. Finally, we have described that RhoA inhibition is a common intracellular mechanisms involved in statins effects. The *in vivo* relevance of these findings was assessed in an experimental model of atherosclerosis in apolipoprotein E deficient mice: Treatment with Atorvastatin increased Smad3 phosphorylation and TRII overexpression, associated to elevated ECM deposition in the VSMCs within atheroma plaques, while apoptosis was not detected.

**Conclusions:**

Statins enhance TGF-β/Smad pathway, regulating ligand levels, receptor, main signaling pathway and cellular responses of VSMC, including apoptosis and ECM accumulation. Our findings show that TGF-β/Smad pathway is essential for statins-dependent actions in VSMCs.

## Introduction

The 3-hydroxy-3-methylglutaryl CoA (HMG-CoA) reductase inhibitors, also known as statins, have been largely reported as very useful drugs in atherosclerosis [1], [2]. They were initially used to treat atherosclerosis because their cholesterol-lowering effects. Nevertheless, multiple pleiotropic beneficial effects have been observed [2]. Statins regulate a huge amount of cellular responses, through the blockade of isoprenoids production and inhibition intracellular signaling systems, including transcription factors, such as nuclear factor-κB (NF-κB), and kinases, like mitogen-activated protein kinases (MAPK) cascade and RhoA/ROCK pathway [3].

Transforming growth factor-β (TGF-β) is a pleiotropic cytokine involved in many human diseases, including cardiovascular disease. TGF-β acts through binding to specific receptors [4], [5], TGF-β receptor type I (TRI), also known as activin-like kinase (ALK), and TGF-β receptor type II (TRII), which are serine/threonine kinases. TRII recruits TGF-β, enabling dimerization with TRI, which transmits TGF-β signaling into the cell [4], [5]. VSMCs present different TGF-β receptor expression profiles in atherosclerotic lesions compared with the normal vessel wall [6]. In normal vessels, TRII is the most abundant receptor. TGF-β through this receptor increases contractile protein expression. In diseased vessels, however, cells dominantly express TRI, as a result of which TGF-β could promote early fatty-streak lesion formation [6]. TGF-β predominantly transmits the signals through cytoplasmic proteins called Smads, which act as transcription factors [4]. In VSMCs, TGF-β1, via ALK5, increases phosphorylation of Smad2 and Smad3, which bind to Smad4. This complex translocates into the nucleus, where it interacts with various transcription factors regulating the expression of TGF-β-responsive genes [7].

A pro-atherogenic role for TGF-β was suspected because of its ability to promote fibrosis [4], [8] and neointima formation, as shown in experimental models of balloon-injury in rats [9], [10]. However, some data suggest a protective role for TGF-β in atherosclerosis [11]. Studies in experimental models of atherosclerosis in mice have shown TGF-β blockade to accelerate plaque formation and its progression toward an unstable phenotype [12]–[14]. TGF-β has protective anti-inflammatory properties due to its immunomodulating effects on key cells in atherosclerosis, including endothelial cells, vascular smooth muscle cells (VSMCs), macrophages, and T cells [11], [14].

An interaction between statins and TGF-β has been suggested. HMG-CoA reductase inhibition increases circulating TGF-β levels and TGF-β synthesis in monocytes [15]. In cardiomyocytes, statins increase TRII expression [16], but there are no data in vascular cells. Recent studies suggests that statin-induced cholesterol lowering effects could increase TGF-β/Smad pathway in endothelial cells[17], [18]. The present study investigates the mechanisms underlying the interaction between statins and TGF-β, and examines whether the beneficial effects of statins in atherosclerosis are attributable to a modulation of the TGF-β/Smad pathway by cholesterol independent mechanisms, through small GTP-ases inhibition. We aimed to demonstrate that statins increase the ability of TGF-β to activate the Smads; being this pathway essential for statin dependent effects on VSMCs, including apoptosis and ECM accumulation.

Studies in VSMCs have shown statins to cause apoptosis. This effect is greater in the presence of Fetal Bovine Serum (FBS) than under serum-free conditions [19], [20]. This apoptotic effect has been subject of study in the latest years, however the primary pharmacological mechanism for statin-induced apoptosis still remains to be completely explained [21]. In the present work we try to elucidate the underlying mechanisms for this process, evaluating the involvement of TGF-β/Smad pathway. The cellular action of statins can be explained by the inhibition of isoprenoids production, which are intermediate components of the cholesterol biosynthetic pathway, such as farnesylpyrophosphate (FPP) and geranylgeranylpyrophosphate (GGPP)[3]. These isoprenoids regulate posttranslational modifications of several proteins, including the small G proteins. In this study we explore how these cellular action regulate TGF-β induced VSMC apoptosis

## Results

### Statins enhance the Smad pathway in cultured vascular smooth muscle cells

**Figure 1:**
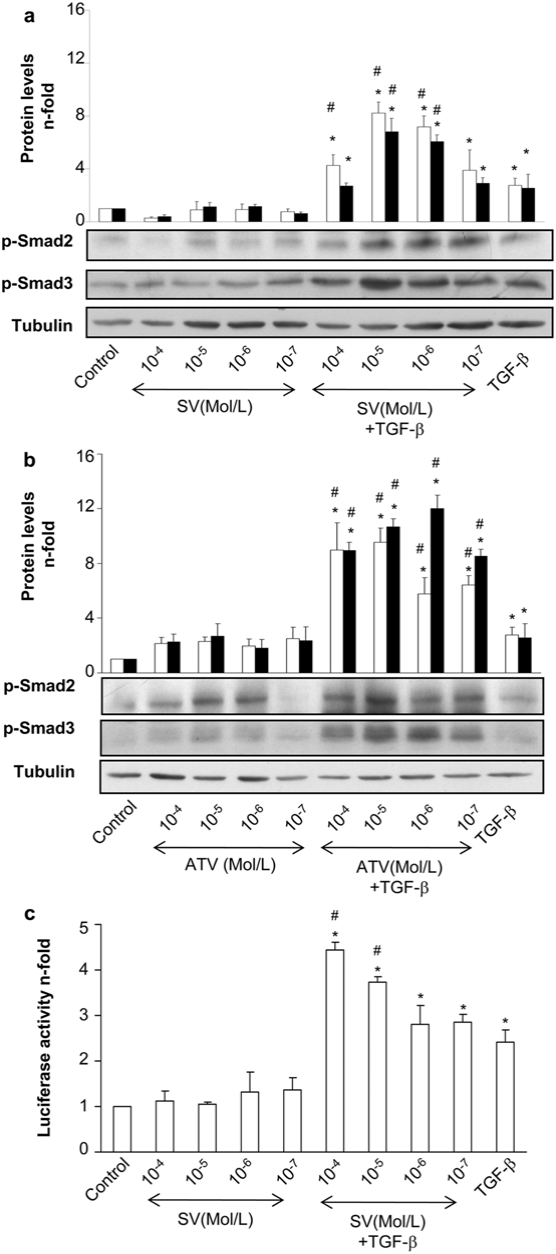
HMG-CoA reductase inhibition enhances the TGF-β/Smad pathway in VSMCs. Serum starved VSMCs were incubated for 48h with several concentrations (10^−4^ to 10^−7^ mol/L) of Simvastatin (SV) (a) or Atorvastatin (ATV) (b). Then, 1 ng/mL TGF-β was added in fresh medium and phosphorylation of Smad2/3 was determined after 20 min of incubation. Lower panel: representative western blot of phosphorylated-Smad2 (p-Smad), p-Smad3, or tubulin (loading control). Upper panel: data of relative protein levels of p-Smad2 (white bars) and p-Smad3 (black bars) expressed as mean±SEM of 4 experiments. Results of p-Smad levels were obtained from densitometric analysis and expressed as ratio p-Smad/tubulin as n-fold over control. *p<0.05 vs. control #p<0.05 vs. TGF-β. c. VSMCs, grown in serum-free medium, were transfected with a Smad-luc reporter vector for 24h. Then, cells were treated for 24h with Simvastatin (SV) or Y-27632. After that, 1 ng/mL TGF-β was added in fresh medium and Smad-dependent transcription was measured after 24h of incubation. *p<0.05 vs. control #p<0.05 vs. TGF-β.

In cultured VSMC, TGF-β induces a rapid activation of Smad pathway, characterized by increased phosphorylation of the regulatory Smads (Smad2/3) after 20 min, binding to Smad4 and nuclear translocation of the complex [4]. The preincubation for 48 h with two statins, simvastatin or atorvastatin, before stimulation with TGF-β for 20 min, significantly increased TGF-β-induced Smad2 and Smad3 phosphorylation in a dose-dependent manner (Figure 1a and b). To confirm this effect, Smad-dependent gene transcription was evaluated using a luciferase-based reporter vector. Pretreatment with simvastatin also increased Smad transcriptional activity in response to TGF-β (Figure 1c), while Smad protein levels were not modified (not show).

### TGF-β/Smad pathway mediates vascular smooth muscle cell death induced by statins

**Figure 2:**
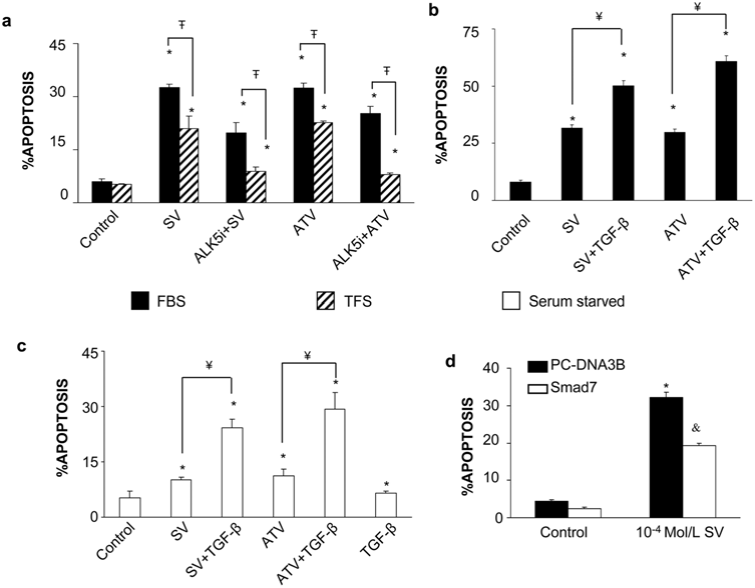
TGF-β mediates statins-induced VSMC apoptosis in 10% FBS. a. VSMCs were incubated with heat inactivated 10% FBS media or with 10% TGF-β-free FBS. Cells were treated with 10^−4^ Mol/L of Simvastatin (SV) or atorvastatin (ATV) for 48h. Some cells were preincubated for 1h with 10^−5^ Mol/L of the TGF-β type I receptor ALK5 inhibitor (ALK5i) before simvastatin or atorvastatin treatment. Black bars represent FBS treated cells; striped bars, cells in TGF-β-free FBS. *p<0.05 vs. control. ∓ p<0.05 vs. TFS. b. TGF-β increases statin-induced VSMC apoptosis. VSMCs were incubated with 10% FBS media. Cells were treated with 10^−4^ Mol/L of Simvastatin (SV) or atorvastatin (ATV) for 48h, some cells were stimulated with 10ng/mL of TGF-β. *p<0.05 vs. control. ¥ p<0.05 vs. 10^−4^ Mol/L SV. c. TGF-β increases statin-induced VSMC apoptosis in serum-free medium. Serum starved cells were treated with 10^−4^ Mol/L of either Simvastatin (SV) or Atorvastatin (ATV) alone or in combination with 1ng/ml TGF-β for 48h in serum-free conditions. *p<0.05 vs. control. ¥ p<0.05 vs. 10^−4^ Mol/L SV. $ p<0.05 vs. 10^−4^ Mol/L SV+TGF-β. d. Smad pathway is involved in Simvastatin induced cell death. Cells grown in 10%FBS were transfected with either control PC-DNA3B plasmid, or with a Smad7 overexpressing vector for 24h. Then cells were treated for 48h with 10^−4^ mol/L Simvastatin. Figure a shows percentage of hypodiploid cells expressed as mean±SEM of 6 experiments. *p<0.05 vs. control &p<0.05 vs. pc-DNA3B transfected cells.

Studies in VSMCs have shown statins to cause apoptosis. This effect is greater in the presence of FBS than under serum-free conditions [19], [20]. We have investigated the involvement of TGF-β/Smad pathway in this process. First, as FBS contains TGF-β, we evaluated whether statin-induced apoptosis is mediated by TGF-β. VSMCs were incubated under two different conditions: FBS or TGF-β-free FBS (TFS), obtained by as described in methods. Treatment with simvastatin or atorvastatin for 48 h in 10% FBS caused a significant increase in cell death that was higher than in 10% TFS (Figure 2a). In 10% FBS, the blockade of TGF-β action, using a specific ALK5 inhibitor (ALK5i), significantly diminished simvastatin-induced VSMC death (Figure 2a). In addition when cells treated with statins were stimulated with 10ng/mL of TGF-β exerted a synergistic apoptotic effect (Figure 2b).

Secondly, we investigated the direct effect of TGF-β on statin-induced apoptosis in serum-free medium. In control experiments, we found the TGF-β content in 10% FBS to be 1 ng/ml (not shown, evaluated by ELISA). The low rate of VSMC apoptosis induced by Atorvastatin or Simvastatin in 0% FBS was dramatically increased when cells were co-incubated with 1ng/ml TGF-β (Figure 2c).

To evaluate the involvement of the Smad pathway in statin-induced cell death in 10% FBS, cells were transiently transfected with a Smad7 overexpressing vector that inhibits TGF-β/Smad-mediated transcriptional effects by interfering with receptor-mediated activation of R-Smad [5]. Smad7 overexpression decreased statin-induced cell death compared to cells transfected with an empty vector (Figure 2d) - suggesting that the Smad pathway drives statin-induced cell death.

### RhoA mediates statin-induced cell death

**Figure 3:**
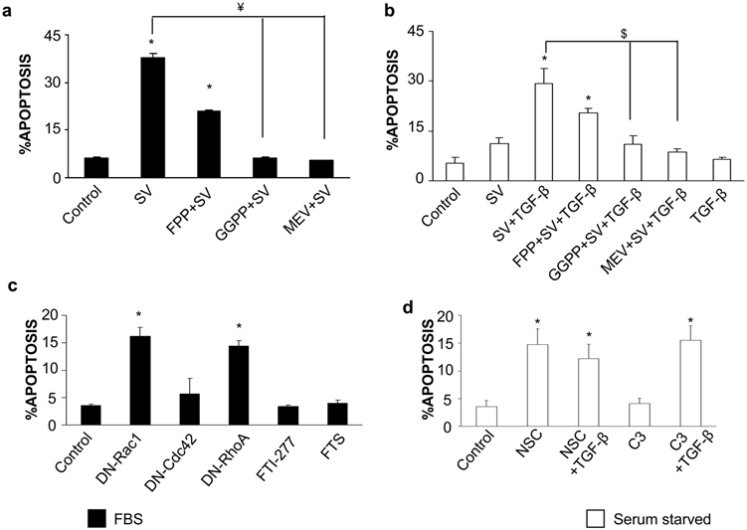
RhoA mediates Simvastatin/TGF-β-induced VSMC death. VSMCs were incubated in 10%FBS medium (black bars: a) or in serum-free conditions (white bars: b). Cells were treated with 10^−4^ mol/L Simvastatin (SV) alone or in combination with 1ng/mL TGF-β for 48h. Some cells were pretreated for 1h with 5 µg/mL GGPP, or FPP, or 10^−4^ mol/L mevalonate (MEV) before Simvastatin treatment. *p<0.05 vs. control. ¥ p<0.05 vs. 10^−4^ Mol/L SV. $ p<0.05 vs. 10^−4^ Mol/L SV+TGF-β c. Cells grown in 10%FBS were either treated with FTI-277, farnesylthiosalicylic acid (FTS), or transfected with a control, DN-Rac1, DN-Cdc42 or DN-RhoA expression vectors and apoptosis was assessed 48h later. *p<0.05 vs. control. d. Cells incubated in serum-free medium were treated with NSC-23766 (50mMol/L) or C3 exoenzyme (10 µg/mL) alone or in combination with 1ng/mL TGF-β for 48h. The graphs show the percentage of hypodiploidic cells measured by FACS and expressed as mean±SEM of 4 experiments. *p<0.05 vs. control.

Several studies suggest that farnesylated proteins, such as Ras, regulate cell growth. In the presence of 10% FBS, several groups, including our own, have shown that small G proteins participate in cell growth regulation by statins[3], [19]. In VSMC several groups have shown that statins inhibited farnesylated proteins[22], [23]. We have observed that FPP only partially restored statin induced apoptosis both in 10% and 0% FBS (figure 3a and b). These results indicate that a farnesylated protein can partially inhibit Statin/TGF-β induced cell death. Treatment with two different farnesyl transferase inhibitors (FTI-277 or FTS) for 48h did not induce VSMC death in 10%FBS (Figure 3c). These data indicate that farnesylated proteins are not involved in statin-induced apoptosis, but they could be involved in VSMC survival. We have observed in VSMCs that preincubation with L-mevalonate, the direct HMG-CoA reductase metabolite, or GGPP, completely abolished simvastatin-induced cell death in both 10% FBS and in TGF-β-treated cells (Figure 3a and b). These data confirm the involvement of a geranylgeranylated, but not a farnesylated protein, in these effects.

We investigated which geranylated protein was involved in the regulation of apoptosis. VSMCs were transfected with dominant negative (DN) expression vectors of the main geranylated proteins Rac-1, Cdc-42 and RhoA. In 10%FBS only DN-Rac-1 and DN-RhoA increased cell death mimicking statin effects (figure 3c). We further determined which GTPase was responsible for the synergistic effect observed with TGF-β/statins. Under serum-free conditions, cells were treated for 48 h with either NSC-23766, a selective inhibitor of Rac1-GEF interaction[24], or with C3 exoenzyme from *Clostridium botulinum,* which inhibits Rho GTPase activity [25]. Rho inactivation increased TGF-β-mediated apoptosis, while blockade of Rac-1 increased apoptosis independently of TGF-β (Figure 3d). This might explain the apoptosis observed with statin treatment alone under serum-free conditions.

**Figure 4:**
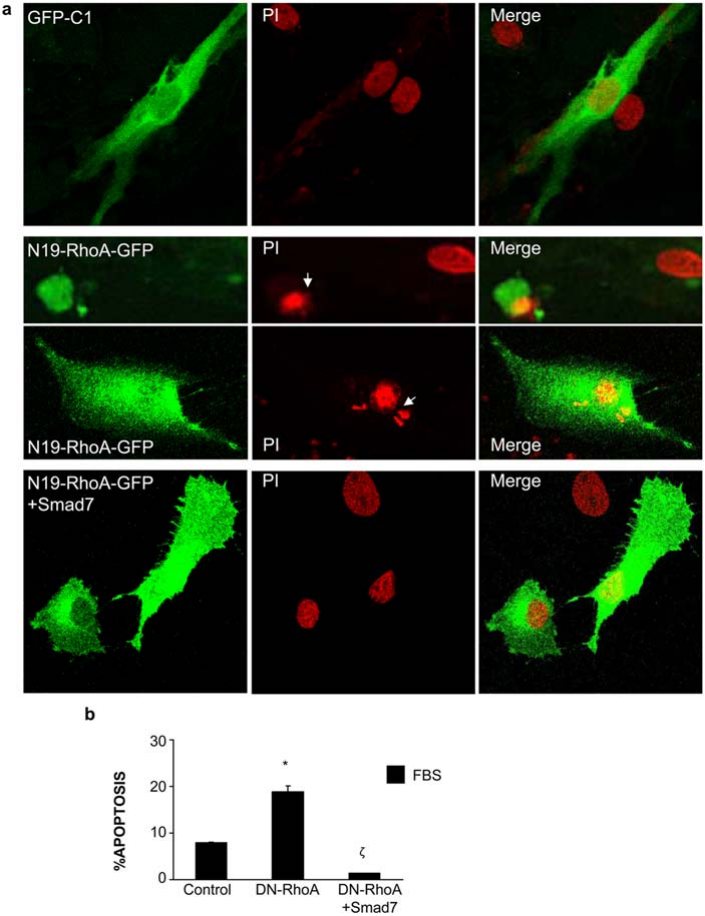
Smad7 inhibits DN-RhoA induced apoptosis. Cells grown in 10%FBS were transfected with a GFP overexpressing vector or with a vector that overexpresses DN-RhoA fused to GFP (N19-RhoA-GFP). Some cells were cotransfected with a Smad7 overexpressing vector. After 48h of transfection apoptosis was evaluated. Figure a shows representative cells (confocal microscopy). Cells were fixed and stained with propidum iodide and mounted in mowiol. Apoptotic nuclei are bright, condensed and present nuclear bodies in the cytoplasm (arrows) and transfected cells appear in green. Figure b shows the percentage of hypodiploid cells expressed as mean±SEM of 4 experiments. *p<0.05 vs. control ζ p<0.05 vs. DN-RhoA transfected cells.

We have further evaluated whether Smad pathway could regulate RhoA-mediated apoptosis. Cotransfection with DN-RhoA and Smad7 markedly suppressed DN-RhoA-induced cell death (Figure 4a and b).

### RhoA/ROCK pathway mediates statin-dependent enhancement of Smad activation

**Figure 5:**
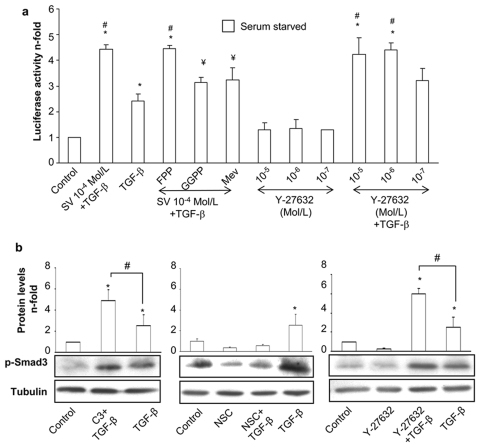
RhoA/ROCK inhibition increases the TGF-β/Smad pathway. a. VSMCs, grown in serum-free medium, were transfected with a Smad-luc reporter vector for 24h. Then, cells were treated for 24h with Simvastatin (SV) or Y-27632. After that, 1 ng/mL TGF-β was added in fresh medium and Smad-dependent transcription was measured after 24h of incubation. Some cells were pretreated for 1h with 5 µg/mL FPP or GGPP or 10^−4^ mol/L mevalonate (Mev) before Simvastatin treatment. Luciferase activity is expressed as mean±SEM of 4 experiments. *p<0.05 vs. control. ¥ p<0.05 vs. SV 10^−4^ Mol/L. b: Serum starved VSMCs were incubated for 48h with C3 exoenzyme (10 µg/mL), NSC-23766 (50mMol/L) or Y-27632 (10^−5^ mol/L), before stimulation with TGF-β for 20 min. Representative western blot and relative protein levels expressed as mean±SEM of 4 experiments. *p<0.05 vs. control. # p<0.05 vs. TGF-β.

We have found that apoptotic effects of statins are driven through inhibition of the small G proteins Rac-1 and RhoA. We decided to explore their role on Smad pathway activation by statins. In VSMCs, incubation with L-mevalonate significantly decreased the effect of statins on TGF-β-induced Smad transcriptional activation, showing the involvement of the direct inhibition of HMG-CoA reductase (Figure 5a). GGPP, but not FPP, inhibited statin-induced Smad enhancement (Figure 5a), suggesting that geranylgeranylated proteins participate in Smad regulation. VSMCs were pretreated for 48 h with either C3 or NSC-23766. RhoA blockade increased Smad3 phosphorylation induced by TGF-β (Figure 5b), whereas Rac-1 inhibition did not increase Smad activation, and even decreased such activation. Pretreatment with Y-27632, an inhibitor of the downstream RhoA mediator ROCK, enhanced Smad-dependent transcription and Smad3 phosphorylation caused by TGF-β (Figure 5b).

### Statins increase TGF-β receptor type II (TRII) and TGF-β expression in cultured vascular smooth muscle cells

**Figure 6:**
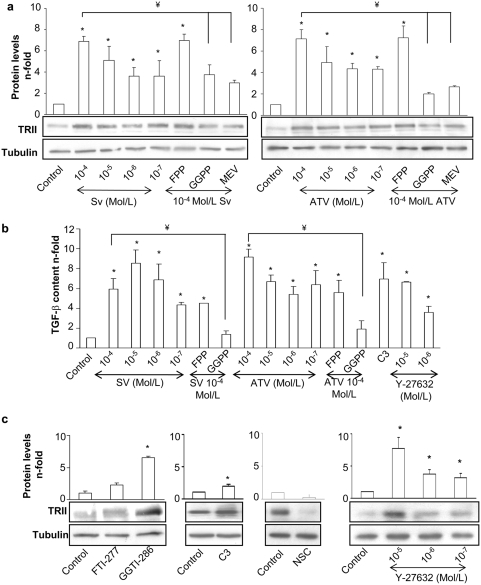
Statins upregulates TGF-β receptor type II (TRII) (a) and TGF-β synthesis in VSMCs (b). Serum starved VSMCs were incubated for 48h with several concentrations (10^−4^ to 10^−7^ mol/L) of Simvastatin (SV) or Atorvastatin (ATV). Some cells were pretreated for 1h with 5 µg/mL FPP or GGPP or 10^−4^ mol/L mevalonate (MEV) before statin treatment. RhoA/ROCK inhibition increases TRII in VSMCs (c) and TGF-β synthesis in VSMCs (b). c. Serum starved VSMCs were incubated for 48h with FTI-277 or GGTI-286(10-5 Mol/L), C3 exoenzyme (10 µg/mL), NSC-23766 (50mMol/L) or Y-27632 (10-5 to 10-7 mol/L). Figures a and c show in the lower panel representative western blot of TRII expression and in the upper panel relative TRII protein levels expressed as mean±SEM of 4 experiments. Figure b shows TGF-β content measured in conditioned media by ELISA. Data are expressed as mean±SEM of n-fold of increase vs unstimulated cells from 4 experiments. *p<0.05 vs. control. ¥ p<0.05 vs. SV 10-4 Mol/L.

We further investigated the effect of statins on TGF-β pathway evaluating its receptor and ligand levels. In VSMCs, incubation with simvastatin or atorvastatin for 48 h significantly increased TRII protein expression and TGF-β secretion in a dose-dependent manner (Figure 6a and b). The upregulation of TRII and TGF-β caused by statins was reversed by pretreatment with either mevalonate or GGPP, but not with FPP (Figure 6a and b). The involvement of the RhoA/ROCK pathway in these processes was also evaluated. Incubation for 48 h with geranylgeranyl transferase inhibitor GGTI-286, but not with farnesyl transferase inhibitor FTI-277 increased TRII protein levels, mimicking the effects of statins (Figure 6c). Inhibition of RhoA and ROCK by C3 and Y-27632, respectively, increased TRII and TGF-β production (Figure 6b and c) in VSMCs, while inhibition of Rac-1 by NSC-23766 decreased TRII levels (Figure 6c).

### Statins enhance the expression of TGF-β dependent ECM regulatory proteins

**Figure 7:**
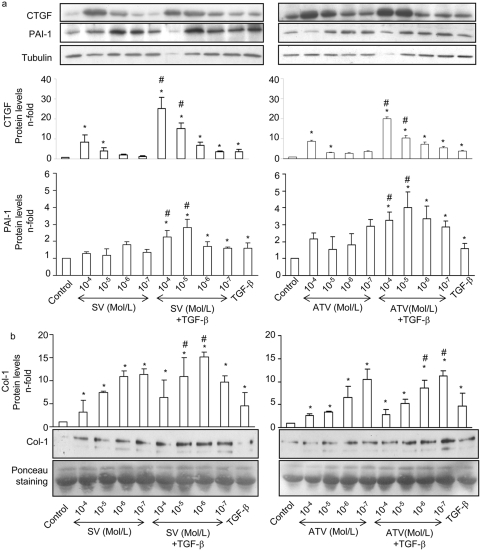
Statins enhance TGF-β dependent ECM regulatory proteins upregulation In VSMCs. a. Serum starved VSMCs were preincubated for 48h with several concentrations (10^−4^ to 10^−7^ mol/L) of Simvastatin (SV) or Atorvastatin (ATV), and then stimulated with 1 ng/mL TGF-β for 24h. These cells were compared with those treated with TGF-β in the absence of statin pretreatment. Figure a shows in the upper panel representative western blot and in the lower panel relative CTGF and PAI-1 protein levels expressed as mean±SEM of 4 experiments. *p<0.05 vs. control. # p<0.05 vs. TGF-β. b. Statins increase Col-1 secretion induced by TGF-β. Serum starved VSMCs were preincubated for 24h with several concentrations (10^−5^ to 10^−7^ mol/L) of Simvastatin (SV) or Atorvastatin (ATV), and then stimulated with 1 ng/mL TGF-β for additional 72h. Conditioned media were analyzed for its Col-1 content by western blot. Figure shows in the lower panel a representative western blot and in the upper panel media the mean±SEM of 4 experiments. *p<0.05 vs. control. # p<0.05 vs. TGF-β.

TGF-β is one of the main profibrotic factors involved in matrix regulation in different cell types and pathological settings. In VSMCs TGF-β, via Smad pathway, regulates ECM-related proteins, including the profibrotic mediator CTGF [4], [26], the inhibitor of ECM degradation, PAI-1, [27], and the main ECM component Type I Collagen (Col-1) [4]. We have found that in VSMCs atorvastatin and simvastatin enhanced TGF-β-dependent CTGF and PAI-1 upregulation in a dose-dependent manner (Figure 7a). Statins also increased the ability of TGF-β to induce Col-1 secretion (Figure 7b).

**Figure 8:**
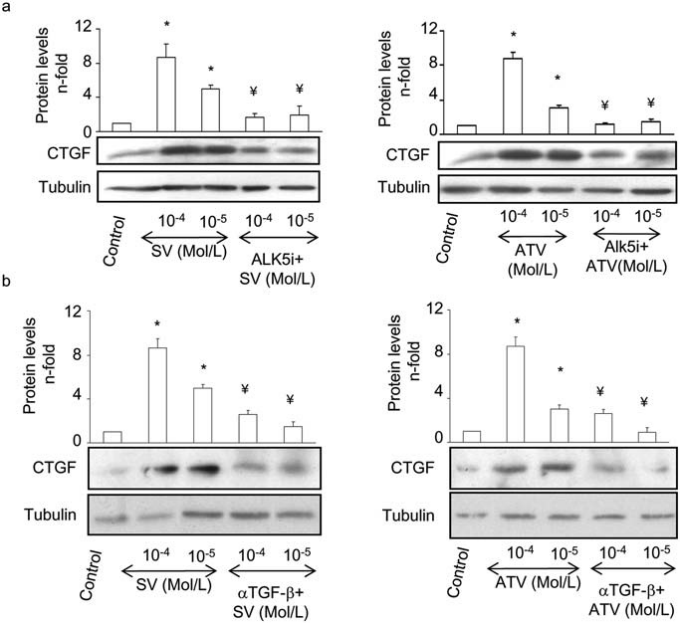
Endogenous TGF-β is involved in Statin-induced CTGF expression. Serum starved VSMCs were preincubated for 72h with highest concentrations of statins a. Some cells were preincubated for 1 h with 10^−5^ mol/L of ALK5i (a) or 10 µg/mL of TGF-β neutralizing antibody (α-TGF-β) (b). Figure shows a representative western blot in the lower panel and in the upper panel the mean±SEM of 4 experiments. *p<0.05 vs. control. ¥ p<0.05 vs. SV 10^−4^ Mol/L.

In the absence of exogenous TGF-β, statins at the doses of 10^−4^ and 10^−5^Mol/L strongly increased CTGF production. We further investigated whether TGF-β is the mediator of statin-induced CTGF overexpression. The blockade of TGF-β with ALK5i or TGF-β neutralizing antibody completely inhibited CTGF production (Figure 8a and b), indicating that endogenous synthesis of TGF-β by statins regulates CTGF overproduction.

### Atorvastatin upregulates TRII and Smad pathway in vivo

**Figure 9:**
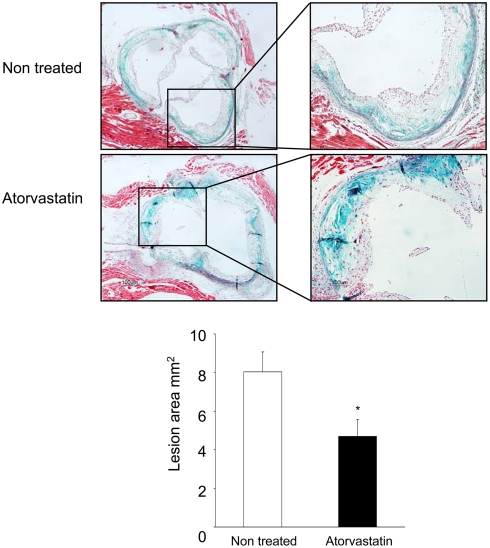
Atorvastatin treatment retards atherosclerosis development. Aortic root samples were stained using Masson tricrome. Lesion areas were measured from the intima to the lumen. Areas were determined using Image Pro-Plus software. Figure shows representative photographs from each group of 8 animals at magnifications of 40× and 100×. Lower panel shows mean±SD of lesion areas in mm^2^. *p<0.05 vs. control.

**Figure 10:**
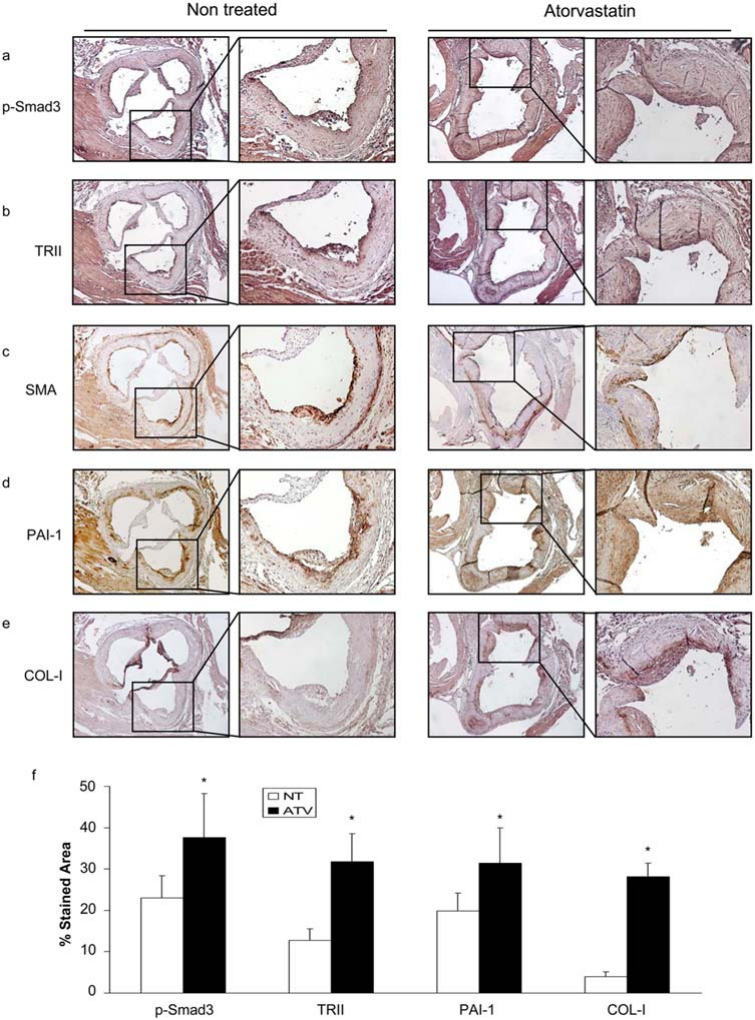
Atorvastatin increases the TGF-β/Smad signaling *in vivo*. ApoE-/- mice of 1 month age were feed with hyperlipidemic diet for 2 months and then randomized in two groups: non treated mice and treated with 5mg/kg/day of atorvastatin for 2 months. Animals were sacrificed and samples were embedded in paraffin. Aortic roots were stained by immunohistochemistry for p-Smad3 (a), TRII (b), Smooth Muscle Actin (SMA) (c), PAI-1 (d), and Type I Collagen (COL-1) (e). Figure shows a representative mouse of each group of 8. magnification 40× and 100×. Panel f shows the quantifications of the histological observations. This was performed by an independent observer, using Metamorph software, and following manufacturer's instructions.

The in vivo relevance of these findings was assessed in an experimental model of atherosclerosis in the apolipoprotein E deficient (ApoE-/-) mice. Treatment with Atorvastatin at moderate dose (5mg/kg/day) ameliorated experimental atherosclerosis, reducing lesion areas of atheroma plaques (Figure 9), as observed in response to statins by many authors. In atherosclerotic lesions atorvastatin increased Smad3 phosphorylation and TRII expression compared to non-treated mice. In serial sections, correlation with Smooth Muscle Actin (SMA) immunostaining was observed, suggesting that TGF-β/Smad pathway is upregulated in VSMCs (Figure 10a–c and f). Increased collagen content has been related to atherosclerotic plaque stabilization [28]. Atorvastatin increased PAI-1 and Col-1 staining in the fibrous cap correlating with SMA positive cells (Figure 10d–f). Previous studies have failed to detect *in vivo* apoptosis in the atheroma plaques [21]. In ApoE-/- mice we have not found apoptosis neither in controls nor in atorvastatin-treated plaques, assessed by immunohistochemistry of cleaved caspase 3 (not shown). These data indicate that Atorvastatin in vivo potentiates Smad pathway in VSMCs leading to elevated ECM protein deposition and amelioration of atherosclerosis, suggesting a novel mechanism of statins involved in plaque stability.

## Discussion

In the present work we report that statins enhance Smad pathway activation by TGF-β. In addition, statins also induced TRII upregulation and increased TGF-β synthesis in VSMCs, causing an amplification of TGF-β-dependent actions. In this sense, statins render VSMCs more susceptible to TGF-β induced apoptosis, and increase TGF-β dependent ECM proteins production.

The Smad pathway is the main signaling system for TGF-β. We have observed that two statins, simvastatin and atorvastatin increased the activation of Smad pathway caused by TGF-β. In VSMCs statins enhanced TGF-β-induced Smad2 and Smad3 phosphorylation and Smad-dependent gene transcription. The Smads interact with transcription factors at the promoters of some genes and regulate their transcription [5]. TGF-β through Smad activation pathway regulates many cellular responses, including cell growth, cell survival, cell differentiation and ECM accumulation [5]. Many studies have demonstrated that statins exert beneficial effects in atherosclerosis. These drugs inhibit multiple intracellular pathways, including NF-κB, the MAPK cascade and RhoA/ROCK signaling - all of them actively involved in the inflammatory process found in aterosclerosis [3]. We have found that statins enhanced the TGF-β/Smad pathway, which is essential for TGF-β-mediated cellular responses.

Smad proteins are nuclear factors that depend on the cellular context to produce their responses [4], [5], [8]. Since statins exert a protective role in atherosclerosis, our data indicate that statins could prepare the cellular context for a protective effect of Smad proteins. In cultured VSMCs we have found that statins increased TGF-β induced apoptosis and ECM production, both process mediated by Smad activation. To find the *in vivo* relevance of these data we have analyzed the effect of atorvastatin in an experimental model of atherosclerosis that has been widely reported to be representative (the ApoE-/- mice model). Atorvastatin increased Smad3 phosphorylation associated to elevated PAI-1 expression, collagen deposition and SMA staining, indicating that the TGF-β/Smad pathway is upregulated in VSMCs within the atheroma plaque and contributes to a more fibrous plaque. In contrast, as discussed later, we failed to detect apoptosis in vivo. Our data in cultured VSMCs show that statins upregulate the profibrotic mediator CTGF, the inhibitor of ECM degradation PAI-1, and collagen production, all of them regulated by Smads [7], [29], [30]. Given that cholesterol has been described to inhibit TGF-β/Smad pathway [17], [18] and that Atorvastatin reduced cholesterol levels, this effect could be attributable to a cholesterol reduction. However as the cholesterol levels remained elevated compared to normal levels (±2 fold, data not shown) and as our *in vitro* results suggest that statin effects are not dependant on cholesterol reduction, it is reasonable to espaculate that *in vivo* effect atorvastatin is likely to be through an inhibition of small GTP-ases.

In atherosclerosis, TGF-β has been considered as a “protective cytokine”, since it plays an important role in maintaining normal vessel wall structure and controls the balance between inflammation and ECM deposition [13], and prevents plaque rupture as shown in human plaques [28]. The loss of this protective effect, attributed to changes in TGF-β receptor profiles, and modulated by local TGF-β levels, contributes to the development of atherosclerosis [31]. We have observed that statins, both in vivo and in vitro increases TRII expression. Differential TGF-β receptors expression in VSMCs from normal vessels and atherosclerotic plaques has been reported [6], [31]. Atherosclerotic plaque-derived cells show almost no TRII expression compared to cells derived from normal vessels - indicating a VSMC phenotype change during atherosclerosis [6], [31]. The observed upregulation of TRII expression in cultured VSMCs suggests that statins could induce phenotype reversion from injured VSMCs to a normal phenotype, showing a potential mechanisms that explain their beneficial effects. We have also found that statins increase TGF-β synthesis in VSMCs. This local production of TGF-β could participate in the beneficial effects of statins. In experimental models, the lack of TGF-β signaling promotes the development of atherosclerotic lesions and unstable plaques [12]–[14]. In human carotid artery plaques, a greater expression of TGF-β in asymptomatic compared to symptomatic lesions has been reported [28]. TGF-β was mainly expressed in the plaque shoulder and was associated with a comparative increase in plaque procollagen and collagen content – thus suggesting that TGF-β may play an important role in the process of plaque stabilization. All these data suggest that statins may increase plaque stability, by increasing collagen content, through TGF-β/Smad pathway enhancement.

Pleiotropics effects of statins have been described in different cell types and pathological conditions. In this sense, RhoA inhibition by statins is responsible for many cellular responses induced by multiple stimuli [3]. However, our findings can not be extrapolated to other pathologies, cells or stimuli. Recently, in a model of cardiac damage induced by Angiotensin II (AngII) infusion, pitavastatin down-regulate Smad signaling pathway and cardiac hypertrophy [32]. In cultured cardiomiocytes statins increase TRII expression [16] indicating that there must be a different regulation of this pathway between cardiomiocytes and VSMCs. We have also observed that atorvastatin ameliorates vascular fibrosis caused by AngII, by a process regulated by RhoA and MAPK activation [33]. Importantly, AngII activates the Smad pathway independently of TGF-β, and regulated by MAPKs, in several cultured cells, including VSMCs [7], [34], and in vivo [35]. It has recently been reported that a PPARγ agonist exert beneficial effects on atheroslcerosis in LDL receptor related protein 1 (LRP1) deficient mice by inhibition of Smad, while TGF-β was reduced [36]. This indicates that probably Smad pathway is activated by a TGF-β independent mechanism. Since statins have been reported to be PPAR agonist [37] it is quite clear that there must be a different regulation between both models. These data suggests that the regulation of this pathway is very complex and depends on the cell type and stimuli studied.

Atherosclerosis is a very complex process that comprises endothelial dysfunction, inflammation, matrix alterations and neointima formation. One of the key steps, probably involved in the origin of atherosclerosis, is VSMC proliferation. Some therapeutic strategies have focused on inhibiting VSMC proliferation [38]. Our in vitro experiments show that under growth-arresting conditions, statins render VSMCs more susceptible to TGF-β induced apoptosis. Many studies have shown that in VSMCs, statins induce apoptosis in the presence of 10% FBS, via the inhibition of small G proteins [19], [20]. This was used as an evidence that statins could increase only proliferative VSMC apoptosis [21]. Our experiments blocking TGF-β action (in TFS and via a specific ALK5 inhibitor) showed TGF-β present in serum to be partly responsible for statin-induced cell death. Moreover, the blockade of Smad activation by Smad7 overexpression diminished statin-induced VSMC apoptosis. These data show VSMC apoptosis caused by statins in the presence of FBS to be mediated by the TGF-β/Smad pathway and the proliferative state is not responsible for that apoptosis. Though statins induce apoptosis in cultured VSMCs as shown here, there are no data showing that statins induce apoptosis in experimental atherosclerosis. It has been suggested that induction of VSMC apoptosis is beneficial to treat atherosclerosis [38]. However, concerns were recently raised that a high rate of VSMC apoptosis induced by diphtheria toxin may be deleterious [39]. In their study Clarke et al indicate that apoptosis of VSMC, in the ApoE-/- mice model of atherosclerosis, increase vascular damage and inflammation, because the high levels of oxidized lipids inhibit the clearance of apoptotic cellular debris by competition with the scavenger receptor. However in our model statin treatment decreases cholesterol levels indicating that clearance of cellular debris is now available. In addition, statins have demonstrated beneficial clinical effects that included amelioration of endothelial dysfunction, inflammation and increased plaque stability [3] [2]. The Clarke's study also shows that huge apoptosis of VSMCs during atherosclerosis development increase instability because the fibrous cap is thinner [40]. In our model atorvastatin increases ECM accumulation what leads to a strengthening of the fibrous cap, while apoptosis was not detectable. Accordingly, it has not been reported that statin increases apoptosis in vivo in any animal models of atherosclerosis. This might be because the dose used for animal models is much lower than the one used in vitro for apoptotic experiments (around 100 times bigger). It could also occur that the most of the apoptosis happens in the early phases of atherosclerosis being highly difficult to detect it. However the *in vivo* contribution of apoptosis to atherosclerosis is beyond the scope of the present work which aimed to determine TGF-β/Smad as an essential pathway driving, at least some, of the statin so called “pleiotropic” effects.

We have evaluated whether the RhoA/ROCK pathway participates in the regulation of TGF-β/Smad pathway caused by statins. In VSMCs, we have found that preincubation with L-mevalonate and GGPP markedly diminishes statin-induced Smad activation and overexpression of TRII and TGF-β, as well as apoptotic effects. Furthermore, these results can be similarly reproduced using RhoA/ROCK inhibitors (by DN-RhoA transfection, C3 pretreatment or ROCK inhibition with Y-27632), but not inhibiting other geranylated GTPases such as Rac1 or Cdc42. This indicates that interaction with TGF-β/Smad signaling can be attributed to RhoA/ROCK inhibition.

**Figure 11:**
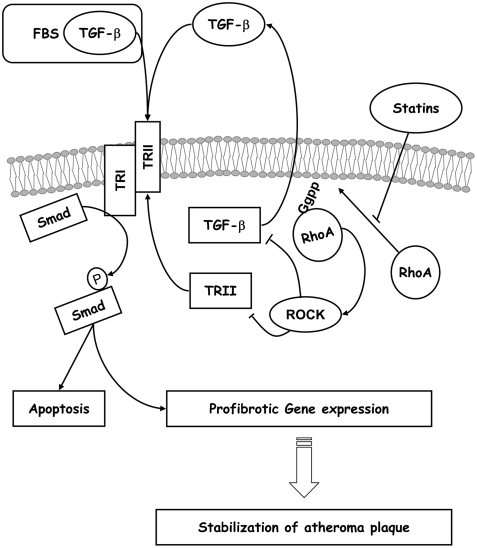
Model for statin action. Proposed model for statin interaction with the TGF-β/Smad pathway in atherosclerosis. In normal conditions TRII and TGF-β expression is regulated by RhoA through ROCK activation. Hyperactivation of RhoA in pathological condition may lead to the downregulation of TRII and TGF-β that characterizes unstable plaques. Statins inhibit prenylation of RhoA, thus increasing TRII expression and TGF- β release. The TRII upregulation allows TGF-β present in serum or induced by statins to activate the Smad pathway and increase ECM proteins production. These effects may promote plaque stability.

Statins are among the best pharmacological options for the treatment of atherosclerosis [1], [2], [41]. Our study reports for the first time that a key factor in their actions is TGF-β. This sheds light upon statin mechanisms of action. To date, attention has focused on pleiotropic effects regarding the inhibition of isoprenylation, but in the present study pleiotropic effects offer a new face. We have shown that such effects are mediated by TGF-β through its main signaling pathway, the Smad proteins, via inhibition of the RhoA/ROCK pathway (Figure 11). This for the first time points to a direct effect of statins in the TGF-β/Smad pathway, that may explain some of the pleiotropic effects described for statin mechanisms of action.

## Methods

### Materials

Cell culture reagents (GIBCO), Simvastatin, FTI-277, GGTI-286, ALK5i, NSC-23766 (Calbiochem), farnesylthiosalicylic acid (FTS) (alexis), Atorvastatin (generously provided by Pfizer), Y-27632 (TOCRIS Cookson Ltd, Bristol, UK), C3 exoezime, farnesylpyrophosphate (FPP) and geranylgeranylpyrophosphate (GGPP), Mevalonate (Sigma-Aldrich) and TGF-β (Peprotech) were used.

TGF-β-free serum (TFS) was obtained by immunoprecipitation. Briefly, decomplemented FBS was incubated with 1 µg/ml of a pan-specific polyclonal TGF-β antibody (R&D) for 1h at 37°C, and then TGF-β was removed by immunocomplex precipitation. TGF-β depletion was determined by ELISA (TGF-β1 immunoassay kit from R&D).

Antibodies employed were: phosphorylated Smad3 (p-Smad3; Abcam for immunohistochemistry, ), pSmad3 kindly donated by Dr. Leof, Mayo clinic, Baltimore (for western blot), phosphorylated Smad2 (p-Smad2), CTGF (Torrey Pines Biolabs) 1∶500, TRII 1∶50, PAI-1 (Santa Cruz biotechnology) 1∶100, type I collagen (Calbiochem) 1∶40, monoclonal anti-α-SMA 1∶200, tubulin (Sigma-Aldrich), and secondary peroxidase-linked antibodies (Amersham).

### Cell cultures

VSMCs from Spragle-Dawley rat thoracic aorta were obtained as previously described [42]. Briefly, Spragle-Dawley rats were obtained from the animal facilities of the Fundacion Jimenez-Dıaz and were treated following Institutional and European guidelines. Subcultured VSMCs from passages 2 to 7 were used in the experiments, showed >99% positive immunostaining against α- SMA antibody and negative for endothelial markers.

### Animal model

Male apolipoprotein E deficient (ApoE-/-) mice were purchased from Jackson Laboratories (Bar Harbor, ME). At 1 month of age, mice diet was changed to a hyperlipidemic diet (21.2% fat (0.15% cholesterol)+16.7% proteins) which was maintained during the whole study in all groups. Four months later, animals were randomized into two groups, non-treated group (n = 8) and mice-treated with atorvastatin, at the dose of 5mg/kg/day (n = 8). After 2 months mice were sacrificed, and different studies were done. Atorvastatin was added to hyperlipidemic diet. During the entire period, the animals had free access to water and food. Experimental mice were killed under anesthesia. All experimental procedures were approved by the Animal Care and Use Committee of our Institution, according to the guidelines for ethical care of the European Community.

### Histology and immunohistochemistry

Tissues were perfused in situ with cold saline followed by removal and overnight fixation for paraffin-embedding. Serial aortic root sections of 3 µm were stained with different techniques. Masson trichrome staining was achieved with Bio-Optica kit (Milan). Lesion areas were measured from the intima to the lumen. Areas were determined using Image Pro-Plus 1.0 software. For immunohistochemistry, endogenous peroxidase activity was blocked with 3% hydrogen peroxide in phosphate buffered saline (PBS) for 30 minutes. The nonspecific binding was blocked with 6% goat or rabbit serum depending on the primary antibody used, in PBS solution for 30 min. Then, slides were incubated with primary antibodies overnight at 4°C. After a PBS rinse, the slides were developed with a biotin-labeled secondary antibody (Amersham). Then, ABComplex/HRP (DAKO) was added and sections were stained with 3,3′-diaminobenzidine (DAKO) and counterstained with hematoxylin. Specificity of the immunostaining was assessed by staining with nonimmune isotype-matched immunoglobulins.

### Protein studies

To quantify protein levels Western blot was done. Cells were homogenized in lysis buffer [(170 mmol/L Tris HCl , 22% glycerol, 2,2% sodium dodecyl sulfate (SDS) with 0,1 mmol/L phenylmethylsulfonyl fluoride, NaF, dithiothreitol, ortovanadate and a protease inhibitor cocktail)] and then separated by SDS-polyacrilamide gel electroforesis. CTGF, PAI-1, TRII and the phosphorylation levels of Smad2/3 were determined in total protein extracts by Western blot. 50 µg of proteins were loaded in each lane; protein content was determined by the BCA method (Pierce, Rockford, IL). The efficacy of protein transfer to the membranes was assessed by Ponceau S staining (not shown). Results of total protein expression were obtained from densitometric analysis using the GS-800 Calibrated Densitometer (Quantity One, Bio-Rad, Spain) and expressed as ratio protein/tubulin as n-fold over control. TGF-β content in conditioned-medium was evaluated by ELISA (TGF-β1 immunoassay kit from R&D).

### Transfection, DNA Constructs, and promoter studies

VSMCs, in 6 well-plate, were transient transfected for 18h with FuGENE (Roche Molecular Biochemicals) and the reporter expression vectors. Smad-dependent promoter activation was evaluated by transfection of 1 µg Smad/luc (kindly donated by Dr. Volgestein, Baltimore) and 0.5 µg TK-renilla as internal control (Clontech). After a 24-h serum starvation step, cells were stimulated for 24 h and assayed for luciferase/renilla. The results are shown as the mean of the experiments of different cell culture preparations, done by triplicate.

To block Smad pathway activation cells were transfected with PcDNA3-FLAG-Smad7 expression vector kindly donated by Dr. Massagué, Memorial Sloan-Kettrin Cancer Center, New York, USA. To demonstrate Smad7 transfection efficacy an anti-FLAG antibody was used (not shown). To inhibit small G-proteins cells were transfected with GFPc1-N19-RhoA, GFPc1-N19-CDC42, and GFPc1-N19-Rac1 which encodes a dominant negative of RhoA fused to GFP, kindly donated by Dr. del Pozo, C.N.I.C, Madrid, Spain. The efficacy of transfection was visualized by confocal microscopy.

### Cell death and apoptosis

For quantification of cell death, cells were seeded in 12-well plates. At defined time points, the cells were harvested by pooling non-adherent cells with adherent cells, which were detached by gentle trypsinization. Apoptosis was quantified by flow cytometry assessment (FACS) of DNA content of 10.000 cells. Pooled attached and detached cells were resuspended in a cell permeabilization buffer containing: 100 µg/mL propidium iodide, 10 µg/mL RNAse A, 0.05% NP-40 in PBS; incubated at 4°C for 1 h; and analyzed on the Cell Quest software. By permeabilizing the cells propidium iodide was allowed access to both dead and live cells. The absolute number of cells with decreased DNA staining, comprising apoptotic cells with fragmented nuclei, was counted.

To assess the typical nuclear changes seen in apoptosis, cells were fixed and stained with propidium iodide. After fixation propidium iodide stains both live and dead cells (more intense). Samples were mounted in Mowiol 40–88 (Sigma) and examined by a laser scanning confocal microscope (Leika).

### Statistical analysis

The autoradiographs were scanned using the GS-800 Calibrated Densitometer (Quantity One, Bio-Rad, Spain). Immunohistochemistry was analized by Metamorph, vesion 6.3r7. Significance was established with SPSS 11.0 software using Tukey, LSD and Bonferroni tests. Differences were considered significant when p<0.05.
